# First-in-human phase 1/1b study to evaluate sitravatinib in patients with advanced solid tumors

**DOI:** 10.1007/s10637-022-01274-y

**Published:** 2022-06-29

**Authors:** Todd Bauer, Byong Chul Cho, Rebecca Heist, Lyudmila Bazhenova, Theresa Werner, Sanjay Goel, Dong-Wan Kim, Douglas Adkins, Richard D. Carvajal, Ajjai Alva, Keith Eaton, Judy Wang, Yong Liu, Xiaohong Yan, Jamie Christensen, Saskia Neuteboom, Richard Chao, Shubham Pant

**Affiliations:** 1 Department of Drug Development, Sarah Cannon Research Institute, Tennessee Oncology, 250 25th Ave North, Ste 100, Nashville, TN 37203 USA; 2 Yonsei University College of Medicine, Seoul, Republic of Korea; 3 Massachusetts General Hospital, Boston, MA USA; 4 University of California San Diego, Moores Cancer Center, La Jolla, CA USA; 5 Huntsman Cancer Institute, The University of Utah, Salt Lake City, UT USA; 6 Albert Einstein College of Medicine, Montefiore Medical Center, New York, NY USA; 7 Seoul National University College of Medicine and Seoul National University Hospital, Seoul, Republic of Korea; 8 Washington University, St. Louis, MO USA; 9 Columbia University Irving Medical Center, New York, NY USA; 10 University of Michigan Medical Center, Ann Arbor, MI USA; 11 University of Washington, Seattle, WA USA; 12 Sarah Cannon Research Institute, Florida Cancer Specialists, Sarasota, FL USA; 13 Mirati Therapeutics, Inc, San Diego, CA USA; 14 University of Texas MD Anderson Cancer Center, Houston, TX USA

**Keywords:** Sitravatinib, Advanced solid tumors, Pharmacokinetics, Adverse events, Objective response rate

## Abstract

Sitravatinib (MGCD516), a spectrum-selective receptor tyrosine kinase inhibitor targeting *TAM* (*TYRO3*, *AXL*, *MERTK*) and split kinase family receptors, has demonstrated preclinical anti-tumor activity and modulation of tumor microenvironment. This first-in-human phase 1/1b study included sitravatinib dose exploration and anti-tumor activity evaluation in selected patients with advanced solid tumors. Primary objectives included assessment of safety, pharmacokinetics and clinical activity of sitravatinib. Secondary objectives included identifying doses for further investigation and exploring molecular markers for patient selection. In phase 1, 32 patients received 10–200 mg, while phase 1b dose expansion comprised 161 patients (150 mg n = 99, 120 mg n = 62). Maximum tolerated dose was determined as 150 mg daily. Dose-limiting toxicity was reported in 4/28 evaluable phase 1 patients (three at 200 mg, one at 80 mg). In phase 1b, 120 mg was defined as the recommended dose due to tolerability. Treatment-related adverse events (TRAEs) were experienced by 174/193 patients (90.2%); grade ≥ 3 TRAEs in 103 patients (53.4%). Most common TRAEs were diarrhea, fatigue, hypertension and nausea; TRAEs led to treatment discontinuation in 26 patients (13.5%) and death in one patient. Sitravatinib was steadily absorbed and declined from plasma with a terminal elimination half-life of 42.1–51.5 h following oral administration. Overall objective response rate was 11.8% in phase 1b, 13.2% in patients with non-small cell lung cancer (NSCLC) and 4.2% in patients with NSCLC with prior checkpoint inhibitor experience. Sitravatinib demonstrated manageable safety and modest clinical activity in solid tumors. NCT02219711 (first posted August 14, 2014).

**Supplementary Information:**

The online version contains supplementary material available at 10.1007/s10637-022-01274-y.

## Introduction

Receptor tyrosine kinases (RTKs) regulate numerous cellular processes including cell proliferation, apoptosis and migration. Aberrant RTK activation is very common in cancer and represents an important therapeutic target for cancer treatment [1]. The utility of RTK-targeted therapies is well-documented in cancers with appropriate genetic alterations, with many targeted therapies now approved worldwide [1].

Sitravatinib (MGCD516) is an orally available small molecule inhibitor targeting closely related spectrum of RTKs, including *TAM* family receptors (*TYRO3*, *AXL*, *MERTK*) and split kinase family receptors (vascular endothelial growth factor receptor 2 [VEGF-R2], *MET*, *RET* and *KIT*). Several sitravatinib targets, such as *TAM* receptors, *MET*, *RET* and *KIT*, are dysregulated in many types of cancer through overexpression or genetic alteration, and contribute to tumor development [2]. Additionally, it is well-known that VEGF and its receptors can drive tumor angiogenesis in cancer [3]. Therefore, by targeting this collection of RTKs, sitravatinib may have meaningful anti-tumor effects.

The potent inhibitory activity of sitravatinib was demonstrated with biochemical half-maximal inhibitory concentration values ranging from 1.5–20 nM against target RTKs, including *AXL*, *MERTK*, VEGF-R, *KIT* and *MET* [4]. Additionally, sitravatinib has demonstrated anti-proliferative effects against solid tumor cells with a variety of phenotypes in vitro, as well as potent anti-tumor activity in xenograft tumor models of lung cancer and sarcoma with RTK dysfunction [4, 5].

Here, we report results for the first-in-human phase 1/1b study of sitravatinib, in patients with advanced solid tumors, including non-small cell lung cancer (NSCLC; clinicaltrials.gov identifier: NCT02219711) [6].

## Materials and methods

### Study design

This study was a multicenter, phase 1/1b clinical trial evaluating the safety, pharmacokinetics (PK) and clinical activity of sitravatinib (free base formulation) in patients with advanced solid tumors. The study comprised two main parts: (1) dose escalation (phase 1); and (2) evaluation of clinical activity in patients selected based on histological diagnoses and/or the presence of defined molecular markers (phase 1b).

In the PK lead-in period, patients received a single oral dose of sitravatinib (10–200 mg) under fasted conditions with at least 200 mL of water, followed by PK sample collection for 3–7 days, depending on emerging PK information. After the PK lead-in period, patients commenced the daily regimen planned for their cohort. Blood samples were collected pre-dose and 0.5 (for 10 mg only), 1, 2, 4, 6, 8, 12, 24, 36, 48, 72 and 168 (for 20–200 mg dose levels) h post-dose from patients following a single oral dose, and at pre-dose, 0.5, 1, 2, 4, 6, 8, 12 and 24 h post-dose following multiple oral doses for the 10–200 mg levels.

The starting dose for the phase 1 dose escalation study was 10 mg once daily (QD). Dose escalation was carried out using the modified toxicity probability interval (mTPI) method [7] with the maximum tolerated dose (MTD) defined as the dose associated with a risk of dose-limiting toxicity (DLT) in 30 ± 5% of patients during the first treatment cycle.

Phase 1b cohorts were organized by diagnosis (renal cell carcinoma [RCC] or castrate-resistant prostate cancer [CRPC]) or by diagnosis of a solid tumor malignancy with a molecular alteration of interest for sitravatinib (such as gene amplification, mutation or rearrangement in *MET*, *AXL*, *RET*, *NTRK, DDR2, KDR, PDGFRA, KIT* or *CBL*).

This study was approved by an institutional review board at each participating site and was conducted in accordance with Good Clinical Practice guidelines, defined by the International Conference on Harmonisation. All patients provided written informed consent.

### Choice of starting dose

The starting dose of 10 mg QD was selected based on non-clinical, 4-week toxicology studies conducted in rats and dogs. In rat studies, 10 mg/kg was the highest dose that did not exceed the severely toxic dose in 10% of the animals (STD_10_). The proposed human dose was based on one-tenth of the STD_10_ in rats corrected for body surface area (mg/m^2^).

### Patient eligibility

Eligible patients were ≥ 18 years old with a histologically confirmed advanced, unresectable or metastatic solid tumor for which standard treatment was not available. Eligible patients had discontinued their most recent prior therapy ≥ 2 weeks before their first dose of study treatment and had recovered from any adverse events (AEs) of their prior therapy back to baseline or grade 1 (excluding alopecia); they also had an Eastern Cooperative Oncology Group performance score (ECOG PS) of 0–2.

Patients included in phase 1b cohorts had a selected diagnosis or tested positive for a designated target tumor molecular marker. The following populations were included: patients with NSCLC with a qualifying molecular alteration in *MET*, *AXL*, *RET*, *NTRK*, *DDR2*, *KDR*, *PDGFRA*, *KIT* or *CBL*; patients with other solid tumor types with a qualifying molecular alteration; patients with clear cell RCC (ccRCC) refractory to angiogenesis inhibitors; and patients with metastatic CRPC (mCRPC) with bone metastases.

Patients with symptomatic or uncontrolled brain metastases and/or with a second active cancer (excluding basal-cell carcinoma or cervical intraepithelial neoplasia) were excluded. For the phase 1b part, patients who had received prior treatment targeting the molecular marker of interest or patients with ccRCC or mCRPC previously treated with cabozantinib were excluded. Further eligibility and discontinuation criteria can be found in the Supplementary Information (Sects. 1.1 and 1.2, respectively).

### Study objectives and assessments

The primary objectives were to characterize the safety profile, PK and clinical activity of sitravatinib. The secondary objectives included exploration of potential pharmacodynamic (PD) markers in blood plasma, identification of doses and regimens of sitravatinib for investigation of clinical activity and exploration of the use of molecular markers for the selection of patients with increased potential for response to sitravatinib.

Safety assessments included evaluation of DLTs, AEs, physical examinations, vital sign measurements, electrocardiogram recordings and laboratory tests. AEs, including laboratory abnormalities, were graded using the National Cancer Institute Common Terminology Criteria for Adverse Events version 4.03 from the day of the first dose of study treatment until ≥ 28 days after the last dose.

PK samples were collected after a single dose during the PK lead-in period and following multiple oral doses during the study. Plasma PK samples were assayed for quantification of sitravatinib. The lower limit of quantification was 0.05 ng/mL. PK parameters were determined using a noncompartmental analysis approach including maximum (peak) concentration (C_max_), time to reach C_max_ following drug administration (t_max_), area under the plasma concentration–time curves (AUCs), apparent total clearance of the drug from plasma after oral administration, apparent volume of distribution during the terminal phase after administration and terminal elimination half-life (t_1/2_). PD effects were examined by analyzing VEGF-A ligand and soluble (s)-VEGF-R2 levels in patients’ plasma samples collected before and after sitravatinib administration.

Clinical activity was assessed by objective response rate (ORR) according to Response Evaluation Criteria In Solid Tumors (RECIST) v1.1. Additional endpoints included duration of response (DoR), progression-free survival (PFS) and overall survival (OS) in phase 1b cohorts of patients based on diagnosis and tumor molecular alterations. An exploratory post-hoc analysis of patients with non-squamous NSCLC who experienced disease progression on prior checkpoint inhibitor (CPI) therapy was performed.

Disease status was evaluated according to RECIST v1.1 at baseline and every three cycles in phase 1, and every two cycles (6-week intervals) in phase 1b. Assessments were performed until objective disease progression was documented or until subsequent anti-cancer therapy started (see Supplementary Information [Sect. 1.3] for details).

### Statistical analyses

The mTPI method [7] was applied for dose escalation. Assumptions applied in establishing the mTPI method included the involvement of up to 30 patients in each regimen explored, a 0.3 probability of DLT at the MTD and an acceptable variance around the MTD of ± 0.05. At least three patients were planned for each cohort, safety permitting.

A DLT was defined as a grade ≥ 4 hematologic abnormality lasting ≥ 4 days; grade 3 thrombocytopenia with clinically significant bleeding; febrile neutropenia; clinically significant grade ≥ 3 non-hematologic AEs not related to underlying malignancy; intolerable grade 2 AEs; or toxicity resulting in an inability to deliver 80% of the dose during the first treatment cycle.

The safety population included all patients who received ≥ 1 dose of sitravatinib. The DLT evaluable population included all phase 1 patients who had taken ≥ 80% of the assigned doses of treatment and were evaluated for toxicity 21 days in the first cycle or had experienced a DLT in cycle 1. The PK evaluable population included all patients with sufficient concentration-time data for PK parameter evaluation. The modified intent-to-treat (mITT) population included all phase 1b patients who received ≥ 1 dose of study drug.

Cohorts of patients defined by tumor molecular markers were evaluated using an optimal Simon 2-stage design. Additionally, an exploratory analysis to describe the ORR in patients with NSCLC was performed.

DoR, PFS and OS were reported descriptively and summarized using the Kaplan–Meier method. DoR was defined as the time from first documentation of objective tumor response (complete response [CR] or partial response [PR]) until first documentation of disease progression per RECIST 1.1 or death (any cause). PFS was defined as the time from first dose of study treatment until progressive disease as defined by RECIST 1.1 or death (any cause). OS was defined as the time from first dose of study treatment until death (any cause).

## Results

### Baseline characteristics

**Fig. 1:**
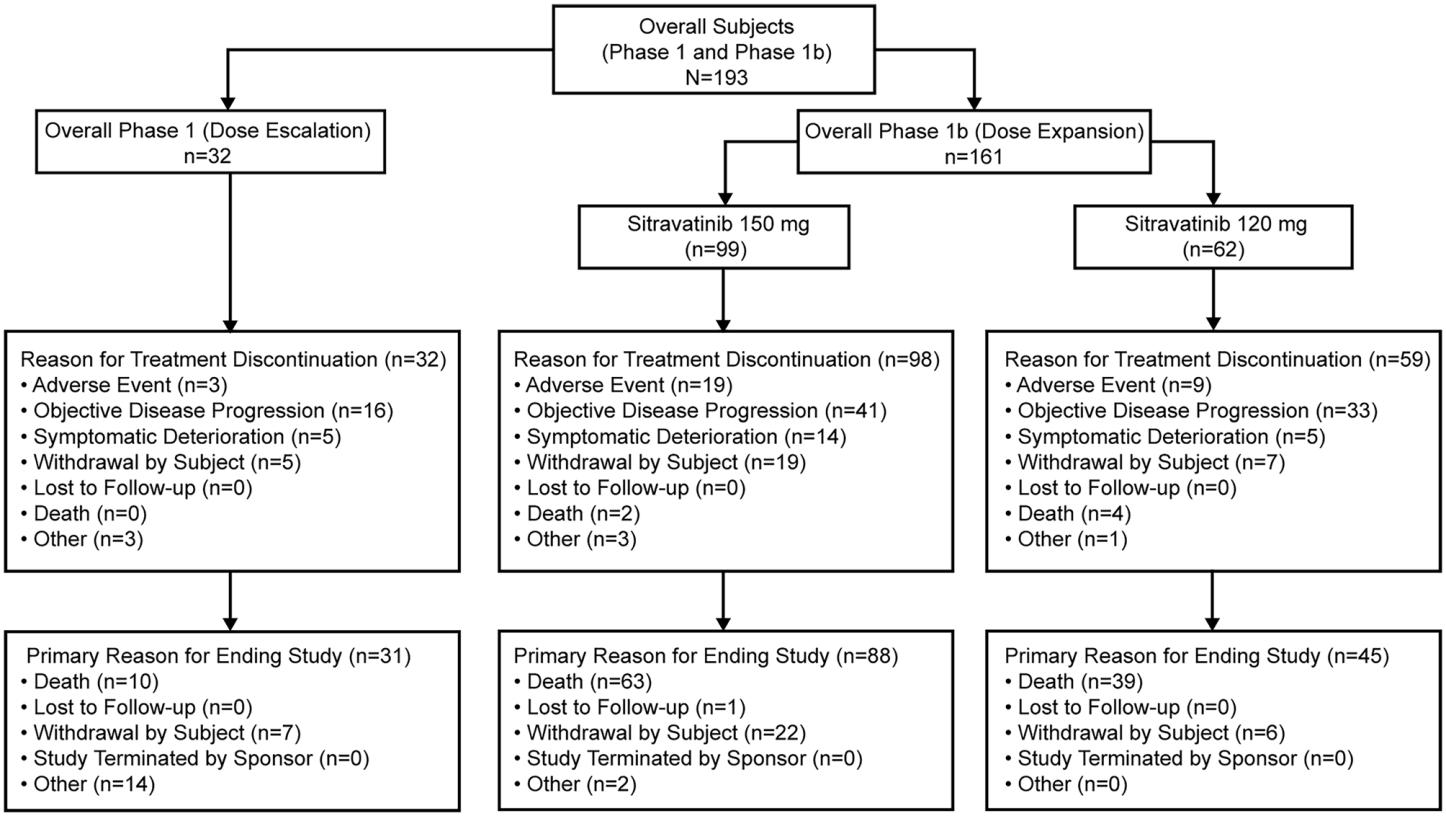
Flow diagram of patients included in this study (N = 193)

Overall, 193 patients received ≥ 1 dose of sitravatinib (safety population). The phase 1 dose escalation cohort comprised 32 patients treated with 10–200 mg, while 161 patients comprised the phase 1b dose expansion cohorts (Fig. 1). In the overall population (n = 193), median age was 65.0 years; 51.8% were male; most patients had ECOG PS 1 (61.7%), had received prior systemic therapy (93.3%) and had mainly NSCLC (29.0%) or RCC (21.2%) (Table 1). Other primary diagnoses are summarized in Supplementary Table S1. For the 53 patients with NSCLC in phase 1b, the histology was adenocarcinoma (n = 45), squamous carcinoma (n = 5) and ‘other’ (n = 3); median age was 66.0 years; 39.6% were male; 60.4% were white and 26.4% were Asian; and 60.4% had ECOG PS 1. In these patients, the median number of prior therapies was two (range, 1–8); 24 patients had received prior immunotherapy, with 20 also having received prior platinum-based chemotherapy. Among the 29 patients who did not receive prior immunotherapy, 24 had received prior platinum-based chemotherapy.

**Table 1:** Patient demographics and baseline characteristics

**Characteristics**	**Phase 1 (n = 32)**	**Sitravatinib 120 mg phase 1b (n = 62)**	**Sitravatinib 150 mg phase 1b (n = 99)**	**Overall (n = 193)**
Age, median (range) years	62.0 (27–85)	65.0 (43–87)	67.0 (36–84)	65.0 (27–87)
Male, n (%)	14 (43.8)	32 (51.6)	54 (54.5)	100 (51.8)
Race, n (%)				
White	24 (75.0)	33 (53.2)	78 (78.8)	135 (69.9)
Asian	2 (6.3)	20 (32.3)	11 (11.1)	33 (17.1)
African American	1 (3.1)	6 (9.7)	5 (5.1)	12 (6.2)
Other	5 (15.6)	3 (4.8)	5 (5.1)	13 (6.7)
ECOG PS, n (%)				
0	16 (50.0)	24 (38.7)	23 (23.2)	63 (32.6)
1	16 (50.0)	33 (53.2)	70 (70.7)	119 (61.7)
2	0	5 (8.1)	6 (6.1)	11 (5.7)
Primary diagnosis, n (%)				
NSCLC	3 (9.4)	25 (40.3)	28 (28.3)^a^	56 (29.0)
Head and neck cancer	0	1 (1.6)	1 (1.0)	2 (1.0)
RCC	3 (9.4)	9 (14.5)	29 (29.3)	41 (21.2)
Hormone-refractory prostate cancer	3 (9.4)	3 (4.8)	10 (10.1)	16 (8.3)
Other^b^	23 (71.9)	24 (38.7)	31 (31.3)	78 (40.4)
Prior therapy, n (%)				
Systemic	31 (96.9)	56 (90.3)	93 (93.9)	180 (93.3)
Radiotherapy	17 (53.1)	30 (48.4)	55 (55.6)	102 (52.8)
Surgery	27 (84.4)	35 (56.5)	79 (79.8)	141 (73.1)
Number of prior systemic regimens, n (%)				
Median (range)	4 (1–10)	3 (1–18)	3 (1–11)	3 (1–18)
1	1 (3.1)	12 (19.4)	13 (13.1)	26 (13.5)
2	6 (18.8)	14 (22.6)	20 (20.2)	40 (20.7)
3	3 (9.4)	11 (17.7)	28 (28.3)	42 (21.8)
4	8 (25.0)	9 (14.5)	13 (13.1)	30 (15.5)
5+	13 (40.6)	10 (16.1)	19 (19.2)	42 (21.8)

*ECOG PS* Eastern Cooperative Oncology Group performance score, *NSCLC* non-small cell lung cancer, *RCC* renal cell carcinoma

^a^One patient was diagnosed with both NSCLC and RCC but was enrolled as a patient with NSCLC

^b^Other’ overall includes soft-tissue sarcoma (6.2%), colon and rectal cancer (5.7%), melanoma (5.2%), breast cancer (2.6%) and various solid tumor types (21.8%). A full breakdown is provided in Supplementary Table S1

### DLTs in Phase 1

Dose levels evaluated among the 32 patients in phase 1 were 10 mg (n = 4), 20 mg (n = 4), 40 mg (n = 5), 80 mg (n = 7), 110 mg (n = 4), 150 mg (n = 4) and 200 mg (n = 4). In phase 1, 4/28 (14.3%) DLT-evaluable patients experienced one DLT each (three at 200 mg and one at 80 mg). Reported DLTs (n = 1 [3.6% of the overall phase 1 population] for each) were intolerable grade 2 fatigue, mucosal inflammation and peripheral sensory neuropathy (all at 200 mg), and grade 3 palmar-plantar erythrodysesthesia (PPE) syndrome (at 80 mg). Thus, 150 mg QD was determined to be the MTD. During phase 1b, the starting dose was decreased to 120 mg QD based on tolerability. Overall, 99 patients in phase 1b received 150 mg sitravatinib as the starting dose; 62 patients received 120 mg sitravatinib as the starting dose.

### Safety

In the safety population (N = 193), the median number of cycles was six and four for patients receiving 150 mg sitravatinib and 120 mg sitravatinib, respectively. In total, 174 patients (90.2%) experienced treatment-related AEs (TRAEs), including 103 (53.4%) who experienced grade ≥ 3 TRAEs (Table 2). The most common TRAEs were diarrhea (50.8%), fatigue (43.0%), hypertension (40.4%) and nausea (30.1%). The most common grade ≥ 3 TRAEs were hypertension (20.7%), diarrhea (10.4%) and fatigue (7.3%). Overall, 26 patients (13.5%) discontinued sitravatinib due to TRAEs; the most common reasons were diarrhea, nausea and fatigue (all in 2.1% of patients). Notably, more patients receiving 150 mg sitravatinib (17.2%) discontinued treatment due to TRAEs compared with patients receiving 120 mg sitravatinib (11.3%). Furthermore, the proportion of patients experiencing serious TRAEs and grade ≥ 3 TRAEs was higher in the 150 mg arm (22.2% and 61.6%, respectively) than in the 120 mg arm (8.1% and 51.6%, respectively; Table 2). Evaluation of TRAEs in patients treated with 120 or 150 mg suggested that 120 mg should be the recommended dose for further exploration. Overall, TRAEs led to treatment modification (dose reduction or treatment interruption) in 120 patients (62.2%), with the most common being diarrhea (17.6%), fatigue (15.0%), hypertension (15.0%) and PPE syndrome (11.9%). Cardiac arrest was the only TRAE leading to death (n = 1, 0.5% of the overall population). This patient was a past smoker with a medical history that included hypothyroidism, mesenteric vein thrombus and hyperlipidemia. Additional safety data are in Supplementary Table S2.

**Table 2:** Summary of TRAEs

**Patients who experienced TRAEs, n of patients (%)**	**Phase 1 (n = 32)**	**Sitravatinib 120 mg phase 1b (n = 62)**	**Sitravatinib 150 mg phase 1b (n = 99)**	**Overall (n = 193)**
Any TRAE	24 (75.0)	58 (93.5)	92 (92.9)	174 (90.2)
Grade ≥ 3	10 (31.3)	32 (51.6)	61 (61.6)	103 (53.4)
Serious	3 (9.4)	5 (8.1)	22 (22.2)	30 (15.5)
Leading to discontinuation	2 (6.3)	7 (11.3)	17 (17.2)	26 (13.5)
Leading to treatment modification	12 (37.5)	42 (67.7)	66 (66.7)	120 (62.2)
Leading to death	0	0	1 (1.0)	1 (0.5)

**Most common TRAEs (≥ 10% of the population) by Preferred Term**	**Any grade**	**Grade ≥ 3**	**Any grade**	**Grade ≥ 3**	**Any grade**	**Grade ≥ 3**	**Any grade**	**Grade ≥ 3**
Hypertension	8 (25.0)	5 (15.6)	24 (38.7)	11 (17.7)	46 (46.5)	24 (24.2)	78 (40.4)	40 (20.7)
Diarrhea	8 (25.0)	2 (6.3)	31 (50.0)	4 (6.5)	59 (59.6)	14 (14.1)	98 (50.8)	20 (10.4)
Fatigue	13 (40.6)	1 (3.1)	21 (33.9)	6 (9.7)	49 (49.5)	7 (7.1)	83 (43.0)	14 (7.3)
PPE syndrome	4 (12.5)	1 (3.1)	13 (21.0)	6 (9.7)	22 (22.2)	4 (4.0)	39 (20.2)	11 (5.7)
Nausea	6 (18.8)	0	14 (22.6)	0	38 (38.4)	5 (5.1)	58 (30.1)	5 (2.6)
Vomiting	6 (18.8)	0	9 (14.5)	0	31 (31.3)	5 (5.1)	46 (23.8)	5 (2.6)
ALT increased	2 (6.3)	0	15 (24.2)	1 (1.6)	18 (18.2)	2 (2.0)	35 (18.1)	3 (1.6)
Decreased appetite	7 (21.9)	0	17 (27.4)	1 (1.6)	27 (27.3)	1 (1.0)	51 (26.4)	2 (1.0)
AST increased	2 (6.3)	0	14 (22.6)	0	20 (20.2)	2 (2.0)	36 (18.7)	2 (1.0)
Stomatitis	3 (9.4)	0	11 (17.7)	1 (1.6)	14 (14.1)	1 (1.0)	28 (14.5)	2 (1.0)
Weight decreased	2 (6.3)	0	7 (11.3)	0	18 (18.2)	2 (2.0)	27 (14.0)	2 (1.0)
Proteinuria	2 (6.3)	0	11 (17.7)	2 (3.2)	9 (9.1)	0	22 (11.4)	2 (1.0)
Rash	4 (12.5)	0	6 (9.7)	2 (3.2)	11 (11.1)	0	21 (10.9)	2 (1.0)
Hypothyroidism	3 (9.4)	0	14 (22.6)	0	16 (16.2)	0	33 (17.1)	0
Dysphonia	2 (6.3)	0	12 (19.4)	0	13 (13.1)	0	27 (14.0)	0
Abdominal pain	0	0	8 (12.9)	0	14 (14.1)	0	22 (11.4)	0
Constipation	2 (6.3)	0	11 (17.7)	0	9 (9.1)	0	22 (11.4)	0
Dry mouth	4 (12.5)	0	7 (11.3)	0	10 (10.1)	0	21 (10.9)	0
Dizziness	2 (6.3)	0	6 (9.7)	0	12 (12.1)	0	20 (10.4)	0

*ALT* alanine aminotransferase, *AST* aspartate aminotransferase, *PPE* palmar-plantar erythrodysesthesia, *TRAE* treatment-related adverse event

### PK and PD analyses

The PK evaluable population comprised 53 patients from the phase 1 and phase 1b cohorts; 40 patients participated in both the PK lead-in and cycle 1 portions, while seven patients participated only in the PK lead-in period and six patients participated only in the cycle 1 PK portion. A few patients in phase 1b receiving 120 mg sitravatinib also participated in the PK lead-in. After single oral administration of 10–200 mg under fasting conditions, sitravatinib was steadily absorbed with a median t_max_ ranging from 3.02–8.87 h and arithmetic mean t_1/2_ ranging from 42.1–51.5 h. After multiple oral administrations of 10–150 mg sitravatinib QD under fasting conditions, median t_max,ss_ ranged between 2.00–8.13 h. At the proposed clinical dose (120 mg QD), the interpatient variability for C_max_ and AUC_τ,ss_ was ~60%.

**Fig. 2:**
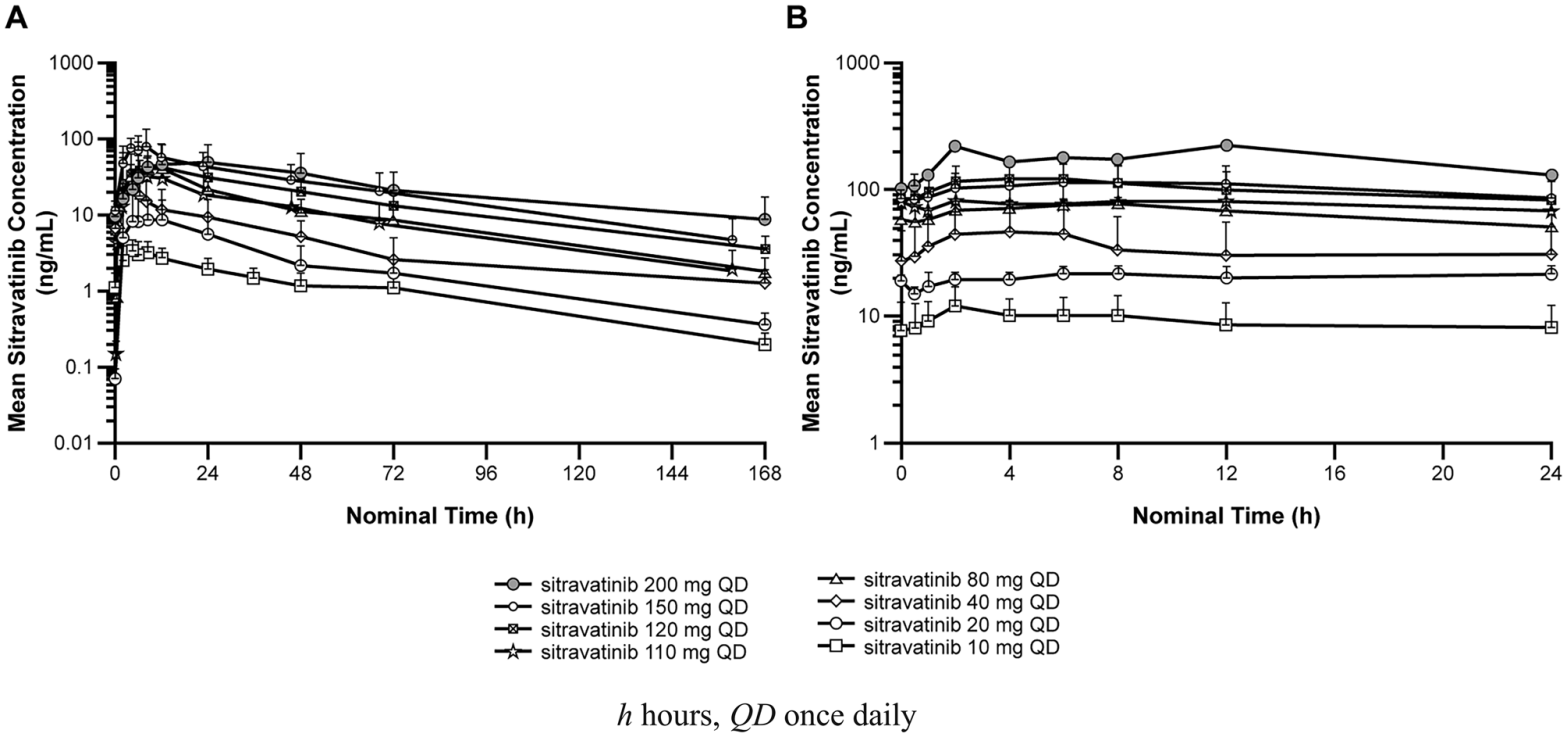
Plasma concentrations of sitravatinib following **A** a single dose and **B** multiple doses over time

Steady-state appeared to have been reached by cycle 1 day 8, and C_max,ss_ and AUC_τ,ss_ accumulation ratios ranged from 1.82–6.89 and 2.13–8.34, respectively. Peak to trough ratios (PTR) in plasma for sitravatinib concentrations at steady state ranged from approximately 1.5–2.1-fold. Sitravatinib exposure (C_max_ and AUCs) appeared to increase in an approximately dose-proportional manner following single- and multiple-dose administration from 10–200 mg, based on a statistical power model where the 95% confidence interval [CI] of the slope estimate for these PK parameters included the value of 1. Figure 2 shows the change in plasma concentration of sitravatinib over time after single and multiple doses. Key PK parameters are in Supplementary Table S3.

PD analysis demonstrated a concentration-dependent modulation of each PD marker with a percent change from baseline for VEGF-A determined as a 200% increase (Supplementary Fig. S1). Based on the EC50 (30.9 ng/mL) from an exposure-response analysis, 120 mg sitravatinib QD is expected to achieve an approximately near maximal effect on the drug target VEGF-R2.

### Clinical activity

In the overall phase 1b mITT population, the ORR was 11.8% (19/161), with all responses being PRs (Table 3). Additionally, phase 1b cohorts were analyzed by diagnosis (RCC or CRPC) or by identification of a tumor molecular alteration of interest (gene amplification, mutation or rearrangement involving *MET, AXL, RET, NTRK, DDR2, KDR, PDGFRA, KIT* or *CBL* gene loci). Responses were observed in patients with RCC and NSCLC, and included patients with tumor *RET* rearrangements, and *MET*, *CBL* and *AXL* alterations (Supplementary Table S4). For patients with NSCLC, the following molecular alterations were reported: *RET* alterations in 24 patients, *MET* alterations in 12 patients, *CBL* alterations in ten patients, Chr4q12 amplification in four patients and *AXL*, *KDR* and *NTRK* alterations in one patient each. The ORR for patients with NSCLC with a molecular alteration of interest was 13.2% (7/53), while that for patients with non-squamous NSCLC and prior CPI experience (exploratory analysis) was 4.2% (1/24) (Table 3).

**Table 3:** Clinical activity in the mITT population

	**Overall (n = 161)**	**NSCLC with molecular alterations (n = 53)**	**Non-squamous NSCLC with prior CPI experience (n = 24)**
ORR, n (%)	19 (11.8)	7 (13.2)	1 (4.2)
CR	0	0	0
PR	19 (11.8)	7 (13.2)	1 (4.2)
SD	88 (54.7)	30 (56.6)	12 (50.0)
PD	28 (17.4)	8 (15.1)	5 (20.8)
NE^a^	26 (16.1)	8 (15.1)	6 (25.0)
DoR, responders, n	19	7	1
6-month KM estimate, % (95% CI)	71.3 (44.0, 87.0)	28.6 (4.1, 61.2)	100 (100, 100)
Median, months (95% CI)	8.2 (4.3, 16.6)	3.0 (1.8, 10.2)	10.2 (NE, NE)
PFS			
6-month KM estimate, % (95% CI)	37.5 (29.2, 45.9)	32.2 (19.2, 46.0)	24.8 (7.8, 46.6)
Median, months (95% CI)	4.3 (3.1, 5.6)	4.3 (2.9, 5.7)	2.9 (1.5, 4.9)
OS			
12-month KM estimate, % (95% CI)	41.3 (32.7, 49.6)	47.6 (32.4, 61.3)	36.8 (16.5, 57.5)
Median, months (95% CI)	10.7 (9.9, 11.9)	11.6 (6.6, 18.2)	5.2 (2.4, 33.8)

Median follow-up, 27.6 months

*CI* confidence interval, *CPI* checkpoint inhibitor, *CR* complete response, *DoR* duration of response, *KM* Kaplan–Meier, *mITT* modified intent-to-treat, *NE* non-evaluable, *NSCLC* non-small cell lung cancer, *ORR* objective response rate, *OS* overall survival, *PD* progressive disease, *PFS* progression-free survival, *PR* partial response, *SD* stable disease

^a^NE patients had no post-baseline scans

In the overall phase 1b mITT population, at the time of data cut-off (median follow-up, 27.6 months), 6-month DoR was 71.3% (95% CI: 44.0, 87.0), with median DoR being 8.2 months (95% CI: 4.3, 16.6) (Supplementary Fig. S2A). In this population, 6-month PFS was 37.5% (95% CI: 29.2, 45.9), with median PFS being 4.3 months (95% CI: 3.1, 5.6) (Supplementary Fig. S2B); 12-month OS was 41.3% (95% CI: 32.7, 49.6), with median OS being 10.7 months (95% CI: 9.9, 11.9) (Supplementary Fig. S2C). Respective clinical activity data stratified by diagnosis and molecular sub-class are in Supplementary Table S4.

## Discussion

Sitravatinib is a potent inhibitor of several RTKs that act as oncogenic drivers, including *RET*, *TAM* receptors and split kinase family receptors. This first-in-human phase 1/1b study demonstrated that sitravatinib had a manageable safety profile with AEs consistent with on-target inhibition and clinical activity was observed in selected populations.

Evaluation of sitravatinib in the phase 1 dose escalation stage resulted in a recommended phase 1b dose of 150 mg daily based on first cycle observations. However, after sequential evaluations of both 150 and 120 mg sitravatinib in phase 1b, 120 mg emerged as the recommended dose for further exploration based on a lower number of discontinuations, serious TRAEs and grade ≥ 3 TRAEs, compared with 150 mg.

Here, the PK profile of sitravatinib was characterized in patients with advanced solid tumor malignancies following single and multiple daily oral administrations from 10–200 mg. Under fasting conditions, sitravatinib was steadily absorbed with a median t_max_ ranging from 3.02–8.87 h and arithmetic mean t_1/2_ ranging from 42.1–51.5 h. At 120 mg QD, the between-patient variability for C_max_ and AUC_τ,ss_ was ~60%. Steady-state appeared to have been reached by cycle 1 day 8 and exposure (C_max_ and AUCs) appeared to increase in a dose-proportional manner. PTR in plasma for sitravatinib concentrations at steady state ranged from approximately 1.5- to 2.1-fold, demonstrating a relatively small difference in steady-state C_max_ and C_min_. The long t_1/2_ and low PTR strongly support a once-daily dosing regimen for sitravatinib. Regarding PD effects, the magnitude of increase in VEGF-A and decrease in s-VEGF-R2 following sitravatinib treatment is consistent with effectively targeting the VEGF-R family and with the effects observed for other agents targeting VEGF-R, including sunitinib, axitinib and cabozantinib [8–10].

Modest clinical activity of sitravatinib was demonstrated in the overall phase 1b population (ORR 11.8%), where almost 60% of patients had received ≥ 3 prior systemic therapies. The ORR for patients with NSCLC with a molecular alteration of interest was 13.2%, which is lower than that reported for next-generation therapies selectively targeting a single kinase, such as *MET* or *RET* [11, 12]. A post-hoc exploratory analysis of patients with NSCLC who experienced disease progression on prior CPI therapy showed that these patients did not gain a clinically meaningful benefit from sitravatinib monotherapy alone (ORR of 4.2%). Overall, these results suggested that sitravatinib, as a monotherapy, did not have significant anti-tumor activity in the analyzed cohorts, including NSCLC. However, sitravatinib is being investigated in combination with CPIs, based on its immunomodulatory role of the tumor microenvironment (TME).

CPI therapy is now established as a breakthrough treatment for various solid tumors, including NSCLC. Although many patients benefit from this treatment, some patients experience disease progression and develop resistance to CPIs through various mechanisms, such as the establishment of an immunosuppressive TME. Previous studies have revealed that targeting *TAM* receptors has an immunomodulatory effect on the TME, particularly involving polarization of tumor-associated macrophage populations [13]. Additionally, it has been demonstrated that targeting VEGF or VEGF-R decreases the number of immunosuppressive cells, such as regulatory T cells and myeloid-derived suppressor cells (MDSCs), in tumor models and patients with cancer [14]. Therefore, the role of sitravatinib in the modulation of the TME has been further explored. Preclinical data demonstrated that sitravatinib could modulate the TME by affecting macrophage polarization through inhibition of the expression of IL-4-stimulated arginase 1 (a marker of M2 polarization) [5]. Additionally, sitravatinib inhibited expression of the M2 markers arginase 1, YM-1 and Fizz-1 upon stimulation with conditioned media from murine cancer cells – a source of *TAM* receptor ligands – and reduced immunosuppressive cell populations, such as MDSCs and M2 macrophages, in vivo [5]. Notably, these changes facilitated a T effector cell response and augmented the effects of anti-programmed death (PD)-1/PD-ligand-1 (anti-PD-1) therapy in these xenograft models [5], and it was therefore hypothesized that the combination of sitravatinib with an anti-PD-1 agent, such as nivolumab, may have a synergistic clinical effect. This hypothesis was tested in a phase 1 window-of-opportunity trial evaluating sitravatinib monotherapy followed by sitravatinib combined with nivolumab in oral cavity cancer [15]. Sitravatinib monotherapy resulted in a less immunosuppressive TME with a reduction in MDSCs and repolarization of macrophages from the M2 to the M1 phenotype [15, 16]. Additionally, sitravatinib followed by the combination with nivolumab for one cycle prior to surgery resulted in tumor reduction for all patients, including one CR [15].

Based on these preliminary data, the anti-tumor efficacy of sitravatinib with CPI therapy has been explored in the MRTX-500 phase 2 study, which evaluated sitravatinib plus nivolumab in advanced NSCLC and indicated encouraging results in patients who had progressed on, or after, prior CPI therapy [17]. These promising data have led to the evaluation of sitravatinib plus nivolumab compared with docetaxel in patients with non-squamous NSCLC in the ongoing phase 3 SAPPHIRE study (NCT03906071) [18]. Additionally, another phase 3 study (NCT04921358) [19] is evaluating sitravatinib plus tislelizumab (a PD-1 inhibitor) compared with docetaxel in patients with locally advanced or metastatic NSCLC.

## Conclusion

In this study, the PK profile of sitravatinib was well characterized, indicating a steady absorption following oral administration and an appropriate t_1/2_ for a once-daily dosing regimen. Sitravatinib had a manageable safety profile and demonstrated modest clinical activity in patients with heavily pretreated advanced solid tumors. Ongoing studies are evaluating sitravatinib in combination with other agents, such as anti-PD-1 inhibitors, in multiple tumor types, including NSCLC.

## Supplementary Information

Below is the link to the electronic supplementary material.

Supplementary file1 (DOCX 379 KB)

## Data Availability

Mirati will honor legitimate requests for clinical trial data from qualified researchers, upon request, as necessary for conducting methodologically sound research. Mirati will provide access to data and clinical study reports (CSRs) for clinical trials for which results are posted on the clinicaltrials.gov registry for products or indications that have been approved by regulators in the US and EU. In general, data will be made available for request approximately 12 months after clinical trial completion. Relevant components of the protocol and statistical analysis plan for this study will also be made available upon request.
