# Supplementary material for: First-in-human phase 1/1b study to evaluate sitravatinib in patients with advanced solid tumors
Source: Invest New Drugs. 2022 Jun 29;40(5):990–1000. doi: 10.1007/s10637-022-01274-y (PMC9395446; doi:10.1007/s10637-022-01274-y)

**Supplementary Appendix**

**First-in-Human Phase 1/1b Study to Evaluate Sitravatinib in Patients with Advanced Solid Tumors**

**Investigational New Drugs**

Todd Bauer^1^, Byong Chul Cho^2^, Rebecca Heist^3^, Lyudmila Bazhenova^4^, Theresa Werner^5^, Sanjay Goel^6^, Dong-Wan Kim^7^, Douglas Adkins^8^, Richard D. Carvajal^9^, Ajjai Alva^10^, Keith Eaton^11^, Judy Wang^12^, Yong Liu^13^, Xiaohong Yan^13^, Jamie Christensen^13^, Saskia Neuteboom^13^, Richard Chao^13^, Shubham Pant^14^

^1^Sarah Cannon Research Institute, Tennessee Oncology, Nashville, TN, USA; ^2^Yonsei University College of Medicine, Seoul, Republic of Korea; ^3^Massachusetts General Hospital, Boston, MA, USA; ^4^University of California San Diego, Moores Cancer Center, La Jolla, CA, USA; ^5^Huntsman Cancer Institute, The University of Utah, Salt Lake City, UT, USA; ^6^Albert Einstein College of Medicine, Montefiore Medical Center, New York, NY, USA; ^7^Seoul National University College of Medicine and Seoul National University Hospital, Seoul, Republic of Korea; ^8^Washington University, St. Louis, MO, USA; ^9^Columbia University Irving Medical Center, New York, NY, USA; ^10^University of Michigan Medical Center, Ann Arbor, MI, USA; ^11^University of Washington, Seattle, WA, USA; ^12^Sarah Cannon Research Institute, Florida Cancer Specialists, Sarasota, FL, USA; ^13^Mirati Therapeutics, Inc., San Diego, CA, USA; ^14^University of Texas MD Anderson Cancer Center, Houston, TX, USA

**Corresponding Author:** Dr Todd M. Bauer, Sarah Cannon Research Institute, Tennessee Oncology, Department of Drug Development, 250 25^th^ Ave North, Ste 100, Nashville, TN, 37203, USA

Telephone number: +1 615-320-5090

Email: [tbauer@tnonc.com](mailto:tbauer@tnonc.com)

# SUPPLEMENTARY METHODS

## 1.1 Patient eligibility

Patients must have met all of the following inclusion criteria to be eligible for enrollment in the study:

1. Histologically confirmed diagnosis of an advanced solid tumor malignancy.
2. Unresectable or metastatic disease for which standard treatment is not available.
3. Age ≥18 years.
4. Life expectancy of at least 3 months.
5. Most recent prior therapy (e.g., chemotherapy, radiation therapy or investigational agent) discontinued at minimum of 2 weeks before first dose date and resolution of toxicities from prior therapy to baseline or grade 1.
6. Recovery from the adverse effects of prior therapy at the time of enrollment to grade ≤1 (excluding alopecia).
7. Eastern Cooperative Oncology Group performance status of 0, 1 or 2.
8. Laboratory values within the screening period:
   1. Absolute neutrophil count ≥1000/mm^3^ (≥1.0 × 10^9^/L) and, in patients enrolled at sites in South Korea, white blood cell count ≥2000/mm^3^ (≥2.0 × 10^9^/L)
   2. Platelet count ≥100,000/mm^3^ (≥100 × 10^9^/L)
   3. Hemoglobin ≥8 gm/dL, in the absence of transfusions for ≥4 weeks
   4. Total bilirubin ≤1.5 × upper limit of normal (ULN) (if associated with liver metastases or Gilbert’s disease, ≤3 × ULN)
   5. Aspartate aminotransferase and alanine aminotransferase ≤2.5 × ULN (if associated with liver metastases, ≤5 × ULN).
   6. Serum creatinine: patient weight >120 lbs (54.4 kg), ≤3.0 × ULN or calculated creatinine clearance ≥ 30 cc/min; patient weight ≤ 120 lbs (54.4 kg), ≤3.0 × ULN and calculated creatinine clearance ≥ 30 cc/min
   7. Urine protein 1+ or less
9. Women of child-bearing potential (WOCBP) or men who are partners of WOCBP agree to use contraception while participating in this study, and for a period of 6 months following termination of study treatment.
10. Completed informed consent process, including signing institutional review board/ethics committee-approved informed consent form.
11. Willing to comply with clinical trial instructions and requirements.

Additional inclusion criteria were required for enrollment into the phase 1b cohort:

1. A selected diagnosis or positive test for a designated target tumor molecular marker in tumor tissue and/or circulating tumor DNA. Target molecular markers will be identified as the study progresses. Positive marker status for the purpose of eligibility must be established using a Sponsor pre-approved method and laboratory. A central laboratory is provided for eligibility screening for patients under consideration for enrollment in a designated phase 1b cohort. If eligibility is established using a local laboratory, tumor specimens, should be available for retrospective confirmation of molecular markers. A representative paraffin-embedded tumor block or a minimum of 12 unstained slides should be available for central laboratory evaluation. Specimens from recent biopsies are preferred, but archival biopsies are acceptable. Biopsies having significant risk should not be performed for the purpose of determining patient eligibility, including but not limited to biopsies of the lung/mediastinum or endoscopic procedures extending beyond the esophagus, stomach or bowel. In the event that the minimum amount of tumor specimen is not available for central review and a new biopsy is not safe, enrollment must be approved by the Sponsor.
2. Evaluable disease, either measurable or non-measurable, in accordance with Response Evaluation Criteria In Solid Tumors (RECIST) v1.1.

Patients presenting with any of the following were not included in the study:

1. Significant cardiac abnormalities within the past 6 months, such as myocardial infarction or congestive heart failure class ≥3 or symptomatic or uncontrolled atrial fibrillation.
2. Prolonged QTc interval on electrocardiogram >480 msec.
3. Patient requires ongoing treatment with concomitant medications known to cause QTc prolongation or known to be sensitive substrates for or substrates with a narrow therapeutic index for P-gp and BCRP transporters.
4. Left ventricular ejection fraction <40%.
5. Patients with squamous cell lung carcinoma and who have either tumor lesions near a major blood vessel or large (>2 cm) cavitating lesions or patients with a history of significant hemoptysis or hemorrhage within 4 weeks of first dose date.
6. Uncontrolled arterial hypertension (>150 mm Hg systolic or >100 mm Hg diastolic) on multiple observations despite standard of care treatment.
7. Symptomatic or uncontrolled brain metastases. Patients with symptoms suggestive of brain metastases must undergo screening computed tomography (CT) or magnetic resonance imaging (MRI) scan of the brain. For patients with history of brain metastases, time elapsed since last cranial radiation must be at least 2 weeks and resolution of acute toxicities to grade 1 or baseline.
8. Another active cancer (excluding basal cell carcinoma or cervical intraepithelial neoplasia/cervical carcinoma in situ). Prior history of cancer is allowed, as long as there is no suspected active disease.
9. Pregnancy. WOCBP must have a negative serum or urine pregnancy test documented within the screening period prior start of study drug.
10. Breast-feeding or planning to breast feed during the study or within 30 days after study treatment.
11. Need for treatment with gastric pH modifying medications including proton pump inhibitors and/or histamine H2 antagonist medications. Patients may switch to use of antacids.
12. Any uncontrolled inter-current illness, active or uncontrolled infection.
13. Any serious illness, medical condition, or other medical history, including laboratory results, which, in the Investigator’s opinion, would be likely to interfere with the patient’s participation in the study, or with the interpretation of the results.
14. Undergone major surgery within 4 weeks of first dose date.
15. Known human immunodeficiency virus seropositivity or active Hepatitis B or C.
16. Any condition (e.g., known or suspected poor compliance, psychological instability, geographical location, etc.) that, in the judgment of the Investigator, may affect the patient’s ability to sign the informed consent and fully comply with all study procedures.
17. Any condition that will put the patient at undue risk or discomfort as a result of adherence to study procedures.

Additional exclusion criteria were required for enrollment into the phase 1b cohort:

1. Prior treatment with a therapy targeting the molecular marker of interest in the phase 1b cohort being considered for enrollment. Specific prior therapies to be excluded based on the patient’s baseline characteristics will be decided following consultation between the Investigator and Sponsor. For example, a prior exposure to onartuzumab (MetMab) would not be considered an automatic exclusion due to differentiated inhibitory activity of sitravatinib for selected *MET* mutant variants and *MET* gene amplification. In contrast, the activity of sitravatinib against *RET* gene rearrangements is not differentiated from other *RET* inhibitors, including cabozantinib or sunitinib, so these prior therapies would be excluded for treatment of *RET* rearrangement-positive lung cancers. Patients having multiple molecular markers of interest may be eligible if prior therapies targeted only one marker. Patients having renal cell carcinoma are expected to have previously received treatment with vascular endothelial growth factor inhibitors and will not be excluded.
2. Clear cell renal cell carcinoma and metastatic castration-resistant prostate cancer patients who received prior treatment with cabozantinib.

Additional exclusion criteria specific to South Korea were also included:

1. Foreseeable need for tooth extraction or dental surgery/procedures that may be associated with osteonecrosis of the jaw.
2. Deep vein thrombosis or other thrombosis requiring ongoing anti-coagulation with vitamin K antagonist. Patients deemed to be clinically stable and receiving maintenance treatment with low-molecular weight heparin are eligible.
3. Need for ongoing treatment with fully therapeutic antiplatelet regimen. Use of prophylactic antiplatelet agents such as low dose aspirin is not excluded.

## 1.2 Discontinuation criteria

Patients were able to discontinue from the study or associated treatment at any time. They may also have discontinued at any time at the discretion of the Investigator or Sponsor for safety, behavioral reasons or inability to comply with the protocol required schedule of study visits or procedures at a given site. Criteria for discontinuation included, but were not limited to:

- Objective disease progression according to RECIST 1.1 as determined by the Investigator (patients who may derive clinical benefit may continue on treatment at the discretion of the Investigator)
- Global deterioration of health status requiring discontinuation
- Adverse event
- Significant protocol violation
- Lost to follow-up
- Refusal for further treatment
- Study terminated by Sponsor
- Death

Reasons for discontinuation from study follow-up may include:

- Completed study follow-up
- Study terminated by Sponsor
- Lost to follow-up
- Refusal for further follow-up for survival
- Death

If a patient discontinued study treatment and/or withdrew consent for disclosure of future information, no further evaluations were performed, and no additional data were collected.

- 1. **Disease assessment**
- Disease assessment was conducted 4 weeks prior to the first study treatment via CT or MRI of the chest and abdomen (including ≥1 measurement extending to the adrenal glands) and ±14 days for on-study disease assessments.

# SUPPLEMENTARY TABLES

## Table S1. Primary diagnosis by preferred term for all enrolled patients

| **Preferred term, n (%)** | **All patients (n=193)^a^** |
| --- | --- |
| Non-small cell lung cancer | 56 (29.0) |
| Renal cell carcinoma | 41 (21.2) |
| Hormone-refractory prostate cancer | 16 (8.3) |
| Head and neck cancer | 2 (1.0) |
| Soft-tissue sarcoma | 12 (6.2) |
| Colon and rectal cancer | 11 (5.7) |
| Melanoma | 10 (5.2) |
| Breast cancer | 5 (2.6) |
| Other  Bladder cancer  Endometrial adenocarcinoma  Ovarian cancer  Pancreatic cancer  Thyroid cancer  Cholangiocarcinoma  Esophageal carcinoma  Malignant neoplasm of unknown primary site  Neuroendocrine tumor  Thymoma  Uterine cancer  Adenoid cystic carcinoma  Basal cell carcinoma  Gastric cancer  Hepatocellular carcinoma  Hürthle cell carcinoma  Osteosarcoma  Ovarian neoplasm  Pancreatic neuroendocrine tumor  Peritoneal carcinomatosis  Renal cell carcinoma  Salivary gland cancer  Small intestine carcinoma | 42 (21.8)  4 (2.1)  4 (2.1)  4 (2.1)  3 (1.6)  3 (1.6)  2 (1.0)  2 (1.0)  2 (1.0)  2 (1.0)  2 (1.0)  2 (1.0)  1 (0.5)  1 (0.5)  1 (0.5)  1 (0.5)  1 (0.5)  1 (0.5)  1 (0.5)  1 (0.5)  1 (0.5)  1 (0.5)  1 (0.5)  1 (0.5) |

^a^Two patients had two types of tumor: one patient had ovarian carcinomatosis and peritoneal carcinomatosis; one patient had breast cancer and pleomorphic sarcoma

**Table S2.** Summary of TEAEs in the overall safety population (n=193)

| **Patients who experienced TEAEs, n of patients (%)** | **Phase 1 (n=32)** | | **Sitravatinib 120 mg  phase 1b (n=62)** | | **Sitravatinib 150 mg  phase 1b (n=99)** | | **Overall (n=193)** | |
| --- | --- | --- | --- | --- | --- | --- | --- | --- |
| Any TEAE  Grade ≥3  Serious  Leading to discontinuation  Leading to treatment modification  Leading to death | 31 (96.9)  20 (62.5)  9 (28.1)  5 (15.6)  13 (40.6)  3 (9.4) | | 60 (96.8)  45 (72.6)  21 (33.9)  12 (19.4)  46 (74.2)  8 (12.9) | | 99 (100.0)  80 (80.8)  49 (49.5)  24 (24.2)  74 (74.7)  10 (10.1) | | 190 (98.4)  145 (75.1)  79 (40.9)  41 (21.2)  133 (68.9)  21 (10.9) | |
| **Most common TEAEs (≥10% of the overall population) organized by System Organ Class and Preferred Term** | **Any grade** | **Grade ≥3** | **Any grade** | **Grade ≥3** | **Any grade** | **Grade ≥3** | **Any grade** | **Grade ≥3** |
| Blood and lymphatic system disorders  Anemia | 7 (21.9)  5 (15.6) | 2 (6.3)  1 (3.1) | 16 (25.8)  10 (16.1) | 7 (11.3)  3 (4.8) | 27 (27.3)  23 (23.2) | 11 (11.1)  8 (8.1) | 50 (25.9)  38 (19.7) | 20 (10.4)  12 (6.2) |
| Cardiac disorders | 3 (9.4) | 2 (6.3) | 6 (9.7) | 2 (3.2) | 15 (15.2) | 6 (6.1) | 24 (12.4) | 10 (5.2) |
| Endocrine disorders  Hypothyroidism | 6 (18.8)  6 (18.8) | 0  0 | 20 (32.3)  19 (30.6) | 0  0 | 27 (27.3)  22 (22.2) | 2 (2.0)  0 | 53 (27.5)  47 (24.4) | 2 (1.0)  0 |
| Gastrointestinal disorders  Abdominal pain  Constipation  Diarrhea  Dry mouth  Nausea  Stomatitis  Vomiting | 25 (78.1)  4 (12.5)  5 (15.6)  13 (40.6)  6 (18.8)  13 (40.6)  4 (12.5)  11 (34.4) | 4 (12.5)  1 (3.1)  0  3 (9.4)  0  1 (3.1)  0  1 (3.1) | 53 (85.5)  14 (22.6)  21 (33.9)  36 (58.1)  7 (11.3)  19 (30.6)  11 (17.7)  12 (19.4) | 12 (9.4)  3 (4.8)  0  4 (6.5)  0  0  1 (1.6)  0 | 89 (89.9)  26 (26.3)  40 (40.4)  67 (67.7)  15 (15.2)  49 (49.5)  14 (14.1)  38 (38.4) | 33 (33.3)  2 (2.0)  0  18 (18.2)  0  7 (7.1)  1 (1.0)  10 (10.1) | 167 (86.5)  44 (22.8)  66 (34.2)  116 (60.1)  28 (14.5)  81 (42.0)  29 (15.0)  61 (31.6) | 49 (25.4)  6 (3.1)  0  25 (13.0)  0  8 (4.1)  2 (1.0)  11 (5.7) |
| General disorders and administration site conditions  Asthenia  Fatigue  Edema peripheral | 21 (65.6)  4 (12.5)  17 (53.1)  7 (21.9) | 3 (9.4)  0  3 (9.4)  1 (3.1) | 38 (61.3)  7 (11.3)  26 (41.9)  6 (9.7) | 8 (12.9)  0  7 (11.3)  0 | 72 (72.7)  9 (9.1)  55 (55.6)  16 (16.2) | 12 (12.1)  0  9 (9.1)  0 | 131 (67.9)  20 (10.4)  98 (50.8)  29 (15.0) | 23 (11.9)  0  19 (9.8)  1 (0.5) |
| Infections and infestations  UTI | 16 (50.0)  3 (9.4) | 5 (15.6)  2 (6.3) | 18 (29.0)  6 (9.7) | 7 (11.3)  1 (1.6) | 40 (40.4)  14 (14.1) | 12 (12.1)  3 (3.0) | 74 (38.3)  23 (11.9) | 24 (12.4)  6 (3.1) |
| Injury, poisoning, procedural complications | 4 (12.5) | 0 | 11 (17.7) | 0 | 20 (20.2) | 2 (2.0) | 35 (18.1) | 2 (1.0) |
| Investigations  ALT increased  AST increased  Blood creatinine increased  Lipase increased  Weight decreased | 16 (50.0)  4 (12.5)  5 (15.6)  3 (9.4)  3 (9.4)  4 (12.5) | 6 (18.8)  1 (3.1)  1 (3.1)  0  1 (3.1) 0 | 34 (54.8)  17 (27.4)  16 (25.8)  3 (4.8)  8 (12.9)  10 (16.1) | 7 (11.3)  1 (1.6)  0  0  3 (4.8) 0 | 66 (66.7)  25 (25.3)  26 (26.3)  15 (15.2)  15 (15.2)  28 (28.3) | 23 (23.2)  3 (3.0)  3 (3.0)  0  10 (10.1) 3 (3.0) | 116 (60.1)  46 (23.8)  47 (24.4)  21 (10.9)  26 (13.5)  42 (21.8) | 36 (18.7)  5 (2.6)  4 (2.1)  0  14 (7.3) 3 (1.6) |
| Metabolism and nutrition disorders  Decreased appetite  Dehydration  Hypokalemia  Hypomagnesemia  Hyponatremia  Hypophosphatemia | 21 (65.6)  15 (46.9)  5 (15.6)  3 (9.4)  3 (9.4)  3 (9.4)  2 (6.3) | 4 (12.5)  1 (3.1)  1 (3.1)  0  0  3 (9.4)  1 (3.1) | 38 (61.3)  21 (33.9)  8 (12.9)  4 (6.5)  4 (6.5)  6 (9.7)  3 (4.8) | 11 (17.7)  2 (3.2)  0  0  0  3 (4.8)  1 (1.6) | 71 (71.7)  38 (38.4)  23 (23.2)  13 (13.1)  17 (17.2)  16 (16.2)  15 (15.2) | 26 (26.3)  1 (1.0)  4 (4.0)  3 (3.0)  0  5 (5.1)  9 (9.1) | 130 (67.4)  74 (38.3)  36 (18.7)  20 (10.4)  24 (12.4)  25 (13.0)  20 (10.4) | 41 (21.2)  4 (2.1)  5 (2.6)  3 (1.6)  0  11 (5.7)  11 (5.7) |
| Musculoskeletal and connective tissue disorders  Back pain  Pain in extremity | 19 (59.4)  8 (25.0)  2 (6.3) | 0  0  0 | 28 (45.2)  6 (9.7)  4 (6.5) | 2 (3.2)  0  0 | 56 (56.6)  18 (18.2)  15 (15.2) | 8 (8.1)  3 (3.0)  2 (2.0) | 103 (53.4)  32 (16.6)  21 (10.9) | 10 (5.2)  3 (1.6)  2 (1.0) |
| Neoplasms benign, malignant, unspecified | 3 (9.4) | 3 (9.4) | 11 (17.7) | 8 (12.9) | 10 (10.1) | 8 (8.1) | 24 (12.4) | 19 (9.8) |
| Nervous system disorders  Dizziness  Headache | 14 (43.8)  5 (15.6)  9 (28.1) | 0  0  0 | 28 (45.2)  10 (16.1)  13 (21.1) | 3 (4.8)  0  1 (1.6) | 50 (50.5)  19 (19.2)  18 (18.2) | 5 (5.1)  0  1 (1.0) | 92 (47.7)  34 (17.6)  40 (20.7) | 8 (4.1)  0  2 (1.0) |
| Psychiatric disorders | 7 (21.9) | 1 (3.1) | 7 (11.3) | 2 (3.2) | 25 (25.3) | 1 (1.0) | 39 (20.2) | 4 (2.1) |
| Renal and urinary disorders  Proteinuria | 9 (28.1)  2 (6.3) | 0  0 | 19 (30.6)  13 (21.0) | 5 (8.1)  2 (3.2) | 23 (23.2)  11 (11.1) | 1 (1.0)  0 | 51 (26.4)  26 (13.5) | 6 (3.1)  2 (1.0) |
| Respiratory, thoracic and mediastinal disorders  Cough  Dysphonia  Dyspnea | 22 (68.8)  12 (37.5)  5 (15.6)  7 (21.9) | 4 (12.5)  0  0  3 (9.4) | 34 (54.8)  7 (11.3)  16 (25.8)  5 (8.1) | 3 (4.8)  0  0  1 (1.6) | 65 (65.7)  15 (15.2)  26 (26.3)  19 (19.2) | 14 (14.1)  0  0  2 (2.0) | 121 (62.7)  34 (17.6)  47 (24.4)  31 (16.1) | 21 (10.9)  0  0  6 (3.1) |
| Skin and subcutaneous tissue disorders  PPE syndrome  Rash | 21 (65.6)  4 (12.5)  5 (15.6) | 1 (3.1)  1 (3.1)  0 | 33 (53.2)  14 (22.6)  9 (14.5) | 8 (12.9)  6 (9.7)  2 (3.2) | 55 (55.6)  23 (23.2)  12 (12.1) | 6 (6.1)  4 (4.0)  0 | 109 (56.5)  41 (21.2)  26 (13.5) | 15 (7.8)  11 (5.7)  2 (1.0) |
| Vascular disorders  Hypertension | 13 (40.6)  11 (34.4) | 6 (18.8)  6 (18.8) | 32 (51.6)  25 (40.3) | 12 (19.4)  12 (19.4) | 61 (61.6)  52 (52.5) | 32 (32.3)  29 (29.3) | 106 (54.9)  88 (45.6) | 50 (25.9)  47 (24.4) |

ALT, alanine aminotransferase; AST, aspartate aminotransferase; PPE, palmar-plantar erythrodysesthesia; TEAE, treatment-emergent adverse event; UTI, urinary tract infection

## Table S3. Pharmacokinetic parameters after single and multiple oral administrations of sitravatinib

| **Single administration** | | | | | | | | |
| --- | --- | --- | --- | --- | --- | --- | --- | --- |
| Parameters^a^ | Sitravatinib dose | | | | | | | |
|  | 10 mg | 20 mg | 40 mg | 80 mg | 110 mg | 120 mg^b^ | 150 mg | 200 mg |
|  | (*n*=4) | (*n*=4) | (*n*=5) | (*n*=7) | (*n*=4) | (*n*=4) | (*n*=15) | (*n*=4) |
| AUC_last_ (h*ng/mL) | 149 (44.6); 4 | 300 (134.4); 4 | 509 (161.8); 5 | 1428 (104.4); 7 | 2030 (84.4); 4 | 1212 (110.5); 4 | 2938 (69.1); 15 | 3296 (89.5); 4 |
| AUC_∞_ (h*ng/mL) | 165 (58.3); 3 | 501 (63.4); 3 | 478 (194.6); 4 | 1540 (104.1); 7 | 2925 (61.0); 3 | 1315 (109.5); 4 | 3562 (63.7); 12 | 4003 (126.1); 3 |
| C_max_ (ng/mL) | 3.54 (39.2); 4 | 5.71 (288.4); 4 | 11.8 (254.1); 5 | 31.7 (120); 7 | 49.1 (62.1); 4 | 23.8 (140); 4 | 69.3 (58.8); 15 | 85.0 (47.5); 4 |
| t_max_^c^ (h) | 5.03  (2.03–11.02); 4 | 8.04  (4.00–10.10); 4 | 4.05  (2.00–24.2); 5 | 6.00  (4.03–8.00); 7 | 3.02  (2.00–10.03); 4 | 8.87  (6.00–10.05); 4 | 6.00  (2.00–22.08); 15 | 5.94  (2.07–8.00); 4 |
| t_1/2_^d^ (h) | 43.6 (26.6); 3 | 47.0 (45.7); 3 | 46.5 (5.3); 4 | 46.2 (25.9); 7 | 44.7 (15.0); 3 | 49.3 (21.2); 4 | 42.1 (19.5); 12 | 51.5 (19.3); 3 |
| CL/F (L/h) | 60.5 (58.3); 3 | 39.9 (63.4); 3 | 83.7 (194.6); 4 | 52.0 (104.1); 7 | 37.6 (61.0); 3 | 91.2 (109.5); 4 | 42.1 (63.7); 12 | 50.0 (126.1); 3 |
| Vz/F (L) | 3720 (51.5); 3 | 2500 (142.5); 3 | 5610 (195.2); 4 | 3360 (106.2); 7 | 2410 (63.0); 3 | 6370 (127.7); 4 | 2510 (60.1); 12 | 3660 (96.4); 3 |
| **Multiple administrations** | | | | | | | | |
|  | Sitravatinib dose | | | | | | | |
|  | Cycle 1 Day 8 | Cycle 1 Day 15 | | | | | | |
| Parameters^a^ | 10 mg QD  (*n*=4) | 20 mg QD | 40 mg QD | 80 mg QD | 110 mg QD | 120 mg QD | 150 mg QD | 200 mg QD |
|  |  | (*n*=3) | (*n*=5) | (*n*=6) | (*n*=3) | (*n*=3) | (*n*=21)^e^ | (*n*=1)^f^ |
| AUC_τ,ss_ (h*ng/mL) | 235  (42.8); 2 | 486  (6.4); 3 | 619  (156.5); 5 | 1218  (104.9); 5 | 2248  (44.5); 3 | 1600  (59.1); 3 | 2181  (46.5); 18 | 4131  (NA); 1 |
| C_max,ss_ (ng/mL) | 11.4  (50.9); 4 | 23.7  (0.9); 3 | 32.5  (173.6); 5 | 69.2  (101.1); 6 | 119  (42.1); 3 | 80.4  (63.1); 3 | 114  (41.4); 20 | 221  (NA); 1 |
| t_max,ss_^c^ (h) | 2.00  (1.97–8.00); 4 | 8.13  (6.00–10.12); 3 | 4.00  (2–8.08); 5 | 4.01  (2.00–8.00); 6 | 2.00  (2.00–6.00); 3 | 4.00  (2.02–10.27); 3 | 7.83  (1.98–10.5); 20 | 10.00  (10.00–10.00); 1 |
| C_av,ss_ (ng/mL) | 9.81  (42.8); 2 | 20.2  (6.4); 3 | 25.8  (156.5); 5 | 50.7  (104.9); 5 | 93.7  (44.5); 3 | 66.7  (59.1); 3 | 90.9  (46.5); 18 | 172  (NA); 1 |
| CL/F (L/h) | 42.5  (42.8); 2 | 41.2  (6.4); 3 | 64.6  (156.5); 5 | 65.7  (104.9); 5 | 48.9  (44.5); 3 | 75.0  (59.1); 3 | 68.8  (46.5); 18 | 48.4  (NA); 1 |
| R_ac_ (AUC) | 3.49  (63.7); 2 | 8.34  (168.8); 3 | 3.53  (30.1); 5 | 2.66  (34.8); 5 | 2.36  (28.0); 3 | 6.44  (65.7); 3 | 2.13  (51.0); 15 | 3.18  (NA); 1 |
| R_ac_ (C_max_) | 3.22  (45.3); 4 | 6.89  (221.7); 3 | 2.76  (36.7); 5 | 2.46  (29.8); 6 | 1.94  (5.9); 3 | 5.17  (64.5); 3 | 1.82  (56.8); 15 | 2.84  (NA); 1 |

^a^Geometric mean (geometric mean CV%); *N*, where *N* = number of observations. ^b^A few patients in Phase 1b receiving 120 mg sitravatinib were also included in the PK lead-in. ^c^Median (min–max); *N*, where *N* = number of observations. ^d^Arithmetic mean (arithmetic mean CV%); *N*, where *N* = Number of observations. ^e^One patient was excluded because the dose administered was different from the planned 150 mg QD dose. ^f^Single patient result.

AUC_0-24_, area under the plasma concentration versus time curve from time 0 to 24 hours; AUC_∞_, area under the plasma concentration-time curve from time zero to infinity; AUC_last_, area under the plasma concentration-time curve from time zero to the time of the last measurable concentration; AUC_τ,ss_, area under the plasma concentration-time curve during the dosage interval; C_av,ss_, average steady-state plasma drug concentration during multiple-dose administration; C_max_, maximum (peak) concentration; C_max,ss_, maximum (peak) steady state concentration; CL/F, apparent total clearance of the drug from plasma after oral administration; CV, coefficient of variation; NA, not applicable; PK, pharmacokinetics; QD, once daily; R_ac_ (AUC), accumulation ratio calculated from AUC_t,ss_ at steady-state (C1D8 and C1D15) relative to and AUC_0-24_ on D1 in the PK lead-in period; R_ac_ (C_max_), accumulation ratio calculated from C_max,ss_ at steady-state (C1D8 and C1D15) relative to and C_max_ on D1 in the PK lead-in period; t_max_, time to reach maximum (peak) concentration following drug administration; t_1/2_, terminal elimination half-life; t_max,ss_, time to reach maximum (peak) steady state concentration following drug administration; Vz/F, apparent volume of distribution during the terminal phase after administration

## Table S4. Clinical activity of sitravatinib in patients with various tumor types and molecular alterations in phase 1b

|  | **RCC**  **(n=38)** | **CRPC**  **(n=10)** | **All molecular alterations**  **(n=113)** | **NSCLC molecular alteration^a^ (n=53)** | **RET rearrangement** | | | **MET alteration**  **(n=17)** | **CBL alteration**  **(n=31)** | **Chr4q12 amplification**  **(n=16)** | **AXL alteration (n=7)** |
| --- | --- | --- | --- | --- | --- | --- | --- | --- | --- | --- | --- |
|  |  |  |  |  | **NSCLC RET rearrangement (n=23)** | **NSCLC KIF5B-RET (n=13)** | **Overall RET**  **(n=31)** |  |  |  |  |
| **Best overall response, n (%)** | | | | | | | | | | | |
| **ORR** | 8 (21.1) | 0 | 11 (9.7) | 7 (13.2) | 4 (17.4) | 1 (7.7) | 5 (16.1) | 2 (11.8) | 2 (6.5) | 0 | 1 (14.3) |
| CR | 0 | 0 | 0 | 0 | 0 | 0 | 0 | 0 | 0 | 0 | 0 |
| PR | 8 (21.1) | 0 | 11 (9.7) | 7 (13.2) | 4 (17.4) | 1 (7.7) | 5 (16.1) | 2 (11.8) | 2 (6.5) | 0 | 1 (14.3) |
| SD | 23 (60.5) | 6 (60.0) | 59 (52.2) | 30 (56.6) | 13 (56.5) | 6 (46.2) | 18 (58.1) | 10 (58.8) | 11 (35.5) | 9 (56.3) | 4 (57.1) |
| PD | 4 (10.5) | 1 (10.0) | 23 (20.4) | 8 (15.1) | 3 (13.0) | 3 (23.1) | 5 (16.1) | 2 (11.8) | 9 (29.0) | 3 (18.8) | 2 (28.6) |
| NE | 3 (7.9) | 3 (30.0) | 20 (17.1) | 8 (15.1) | 3 (13.0) | 3 (23.1) | 3 (9.7) | 3 (17.6) | 9 (29.0) | 4 (25.0) | 0 |
| **Duration of response** | | | | | | | | | | | |
| **Responders, n** | 8 | NA | 11 | 7 | 4 | 1 | 5 | 2 | 2 | NA | 1 |
| **6-month KM estimate, % (95% CI)** | 100.0 (100.0, 100.0) | NA | 53.0  (20.9, 77.3) | 28.6  (4.1, 61.2) | 25.0  (0.9, 66.5) | 100.0  (100.0, 100.0) | 40.0  (5.2, 75.3) | NA | 50.0  (0.6, 91.0) | NA | NA |
| **Median, months  (95% CI)** | 13.2  (6.8, NE) | NA | 7.0  (1.9, 15.2) | 3.0  (1.8, 10.2) | 2.3  (1.8, 10.2) | 10.2  (NE, NE) | 2.8  (1.8, 10.2) | NR  (3.0, NE) | NR  (4.3, NE) | NA | NR |
| **Progression-free survival** | | | | | | | | | | | |
| **6-month KM estimate, % (95% CI)** | 62.6  (43.5, 76.9) | 50.0  (11.1, 80.4) | 28.5  (19.6, 38.0) | 32.2  (19.2, 46.0) | 40.3  (19.5, 60.4) | 36.4  (11.2, 62.7) | 39.5  (21.8, 56.7) | 16.4  (2.8, 40.0) | 12.3  (3.1, 28.3) | 23.8  (5.8, 48.5) | 71.4  (25.8, 92.0) |
| **Median, months  (95% CI)** | 9.5  (4.3, 11.7) | 5.8  (2.1, NE) | 3.6  (2.7, 4.4) | 4.3  (2.9, 5.7) | 5.7  (3.0, 13.5) | 5.7  (1.5, 13.5) | 5.3  (3.0, 6.7) | 2.9  (1.9, 4.3) | 2.0  (1.3, 4.1) | 2.7  (1.3, 4.4) | NR  (1.2, NE) |
| **Overall survival** | | | | | | | | | | | |
| **12-month KM estimate, % (95% CI)** | 60.1  (41.3, 74.6) | 18.2  (0.8, 54.5) | 36.1  (26.3, 45.9) | 47.6  (32.4, 61.3) | 73.2  (46.6, 88.1) | 79.5  (39.3, 94.5) | 66.5  (43.3, 82.0) | 27.1  (8.4, 50.2) | 20.2  (7.4, 37.3) | 35.2  (13.3, 58.2) | 26.8  (1.3, 67.0) |
| **Median, months  (95% CI)** | 29.3  (10.8, 33.4) | 10.1  (2.8, 23.2) | 9.9  (7.1, 10.9) | 11.6  (6.6, 18.2) | 27.2  (10.9, NE) | 27.2  (10.9, 33.8) | 18.2  (10.9, 33.8) | 7.3  (3.5, 13.9) | 5.0  (3.2, 9.9) | 9.5  (3.6, 18.1) | 10.0  (2.2, NE) |

^a^NSCLC molecular alterations included: *RET* alterations (*n*=24), *MET* alterations (*n*=12), *CBL* alterations (*n*=10), Chr4q12 amplification (*n*=4), and *AXL*, *KDR* and *NTRK* alterations (*n*=1 each). CI, confidence interval; CR, complete response; CRPC, castrate-resistant prostate cancer; IQR, interquartile range; KM, Kaplan–Meier; NA, not available; NE, not estimable; NR, not reached; NSCLC, non-small cell lung cancer; ORR, objective response rate; PD, progressive disease; PR, partial response; RCC, renal cell carcinoma; SD, stable disease

**SUPPLEMENTARY FIGURES**

## Fig. S1 Exposure pharmacodynamic soluble biomarkers VEGF-A (A) and s-VEGF-R2 (B) percent change from baseline

**A**


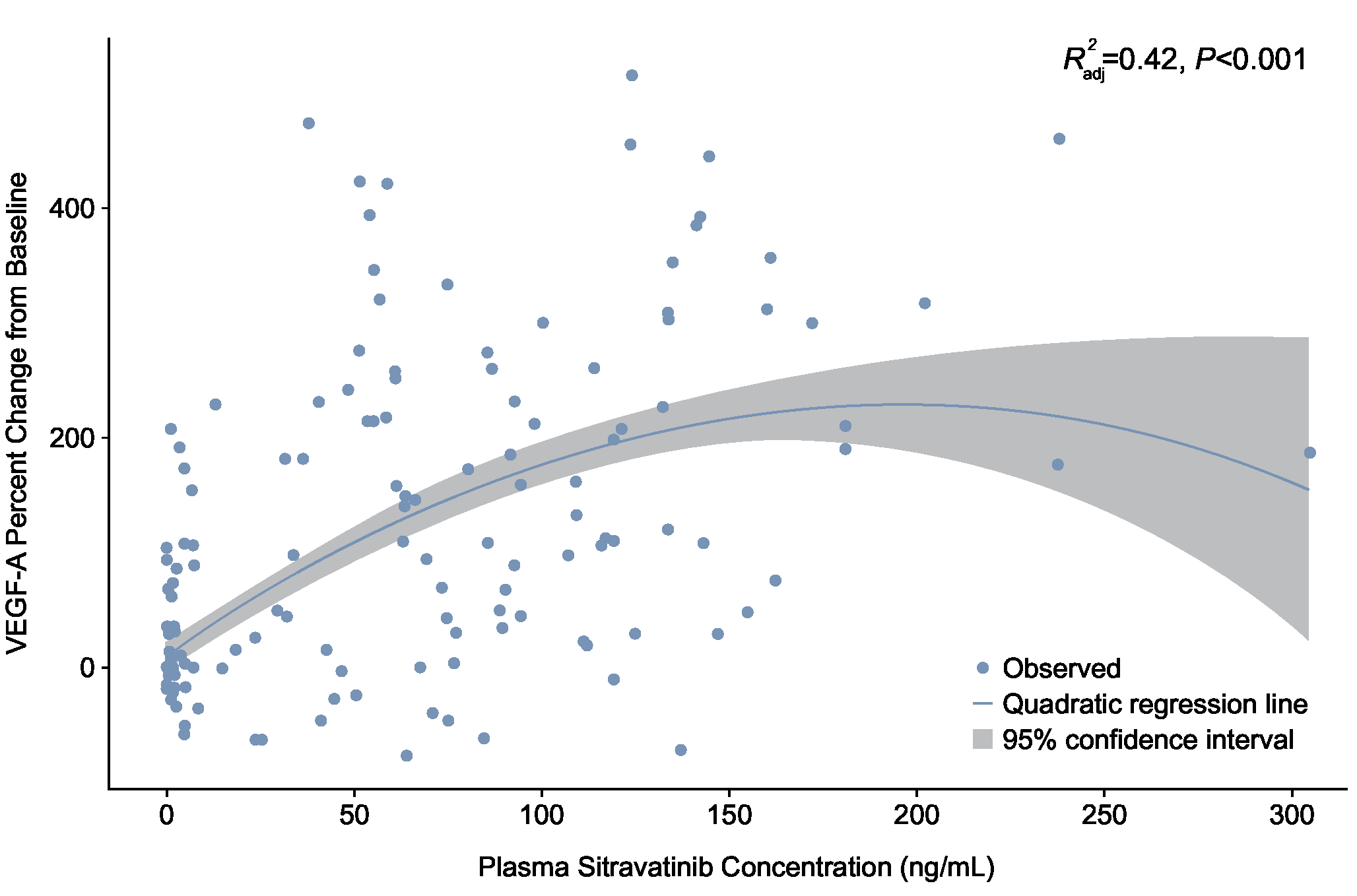


**B**

**
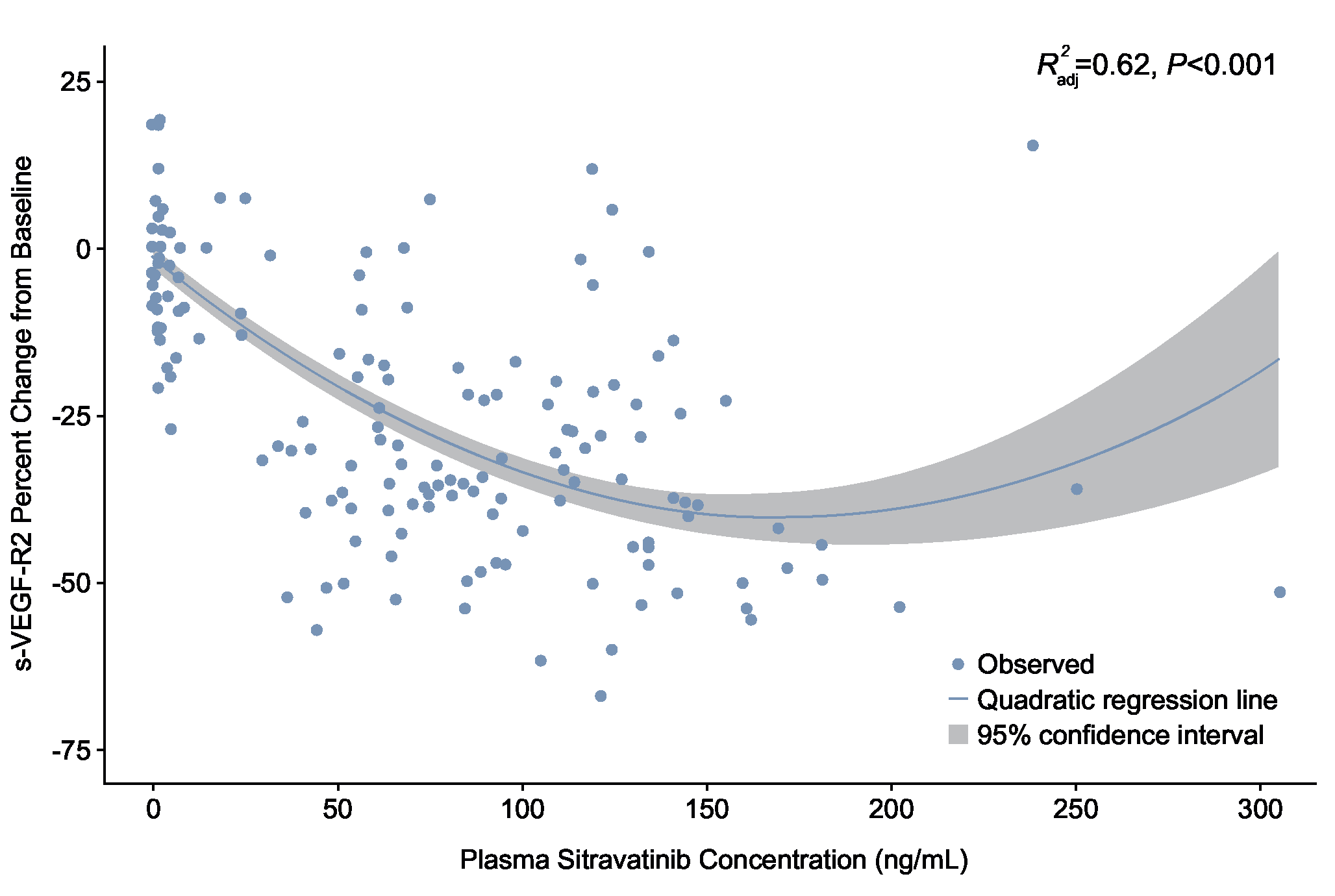
**

## R^2^_adj_, R-squared adjusted; s-VEGF-R2, soluble vascular endothelial growth factor receptor 2; VEGF-A, vascular endothelial growth factor A.

## Fig. S2 Overall phase 1b population efficacy: duration of response (A), progression-free survival (B) and overall survival (C)


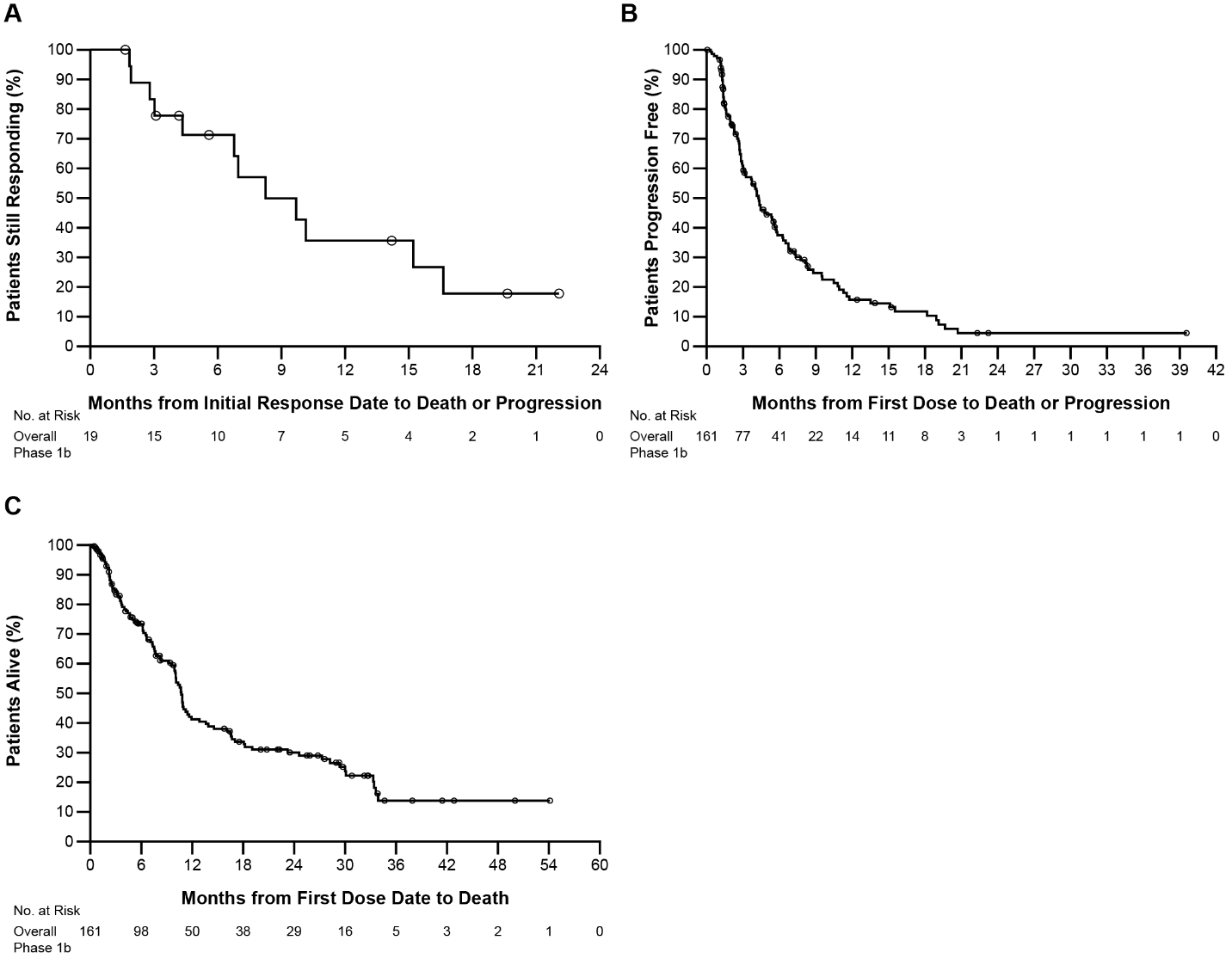

Supplement: Supplementary file 1 — Supplementary file1 (DOCX 379 KB) [file 10637_2022_1274_MOESM1_ESM.docx]
